# Editorial: Microbial regulatory mechanisms in remediation of industrial wastewater and contaminated soils

**DOI:** 10.3389/fmicb.2025.1585585

**Published:** 2025-03-25

**Authors:** Rui Liu, Yuling Zhang, Weitao Liu, Jianjun Chen

**Affiliations:** ^1^School of Ecology and Environmental Sciences, Institute for Ecological Research and Pollution Control of Plateau Lakes, Yunnan University, Kunming, China; ^2^College of New Energy and Environment, Jilin University, Changchun, China; ^3^College of Environmental Science and Engineering, Nankai University, Tianjin, China; ^4^Department of Environmental Horticulture, Mid-Florida Research and Education Center, Institute of Food and Agricultural Sciences, University of Florida, Apopka, FL, United States

**Keywords:** bacteria, biodegradation, bioremediation, biosorption, heavy metals, industrial wastewater, organic pollutants

With the rapid increase in population and the expansion of industrialization, the release of industrial wastewater and other pollutants into the environment is increasing significantly, posing a serious threat to environmental quality and human health. In recent decades, various technologies have been developed to remove or minimize heavy metals and refractory organic pollutants in contaminated water and soil. Among these, microbes play an important role in the remediation process. Thus, a deeper understanding of microbial regulatory mechanisms will provide a basis for improving remediation efficiency.

This Research Topic aimed to collect full-length articles, reviews, and communications addressing the current research progress in microbial remediation processes, co-metabolic mechanisms, the search for highly efficient degrading microorganisms, and their application in industrial wastewater treatment and contaminated soil remediation. Eight articles were accepted into this Research Topic, which included studies on bacteria and fungi in either the adsorption or stabilization of heavy metals in soils and bacteria and fungi in the bioremediation of organic pollutants.

Heavy metals are persistent pollutants in water and soil. Remediation of heavy metals through microbial methods is a fast, simple, and easily adaptable approach. Microbes can remediate them through sorption, accumulation, transformation, and mineralization. Biosorption is a metabolically independent process that mainly relies on microbial surface functional groups as binding sites. To optimize the biosorption of Pb on *Bacillus subtilis*, El-Sharkawy et al. analyzed a definitive screening design (DSD) and an artificial neural network (ANN) in the sorption of Pb. The authors found that the ANN model provided satisfactory results for predicting Pb biosorption. The Langmuir isotherm and pseudo-second-order models with R^2^ of 0.991 and 0.999 were suitable for the biosorption data, predicting a monolayer adsorption and chemisorption mechanism, respectively.

Superalloy electrolytes are acidic solutions used for machining superalloys. Such solutions generally contain significant quantities of precious metals. Yang et al. evaluated five fungal strains for the adsorption of heavy metal ions. *Paecilomyces lilacinus* was identified as the most effective strain, adsorbing 37.1, 64.4, 47.9, 41.6, and 25.4% of Co, Cr, Mo, Re, and Ni, respectively, in 1 h, at a pH of 5. Based on Fourier transform infrared spectroscopy, the fungal surface functional groups: hydroxyl (-OH), amino (-NH_2_), amide (-CONH_2_), carbonyl (-C = O), carboxyl (-COOH), and phosphate (${\text{PO}}_{4}^{3-}$) participated in the adsorption process.

Biomineralization is another mechanism that microbes use to remediate heavy metals. Zhu et al. investigated the role of phosphate-solubilizing bacteria (PSB) and urease-producing bacteria (UPB), comprising six *Bacillus* strains, in stabilizing heavy metals. PSB can dissolve insoluble phosphorus, leading to the precipitation of metal ions; UPB can hydrolyze urea to produce carbonate ions that contribute to the formation of carboxylate metal complexes. Synergistic effects were noted using both PSB and UPB strains, resulting in stabilization rates of more than 92% for Cu, Zn, Cd, and Pb, respectively. The biomineralized products were mainly carbonate and phosphate precipitates.

Cyanobacteria are photoautotrophic species that possess remarkable abilities to remediate heavy metals. In addition to biosorption, this group of bacteria can take up metals from the environment and accumulate them inside the cells by detoxification, compartmentation, and transformation because they lack extensive internal-bound membranes. Lourembam et al. reviewed the potential of cyanobacteria in the biosorption, bioaccumulation, and sequestration of heavy metals on the surface and inside of cells, providing the current status and future perspectives on the use of cyanobacteria to remediate heavy metals.

Refractory organic pollutants are another group of contaminants in the environment. Microbes are the most effective agents for remediating these pollutants as they can break them down into less toxic or harmless compounds. Li et al. reported that *Candida tropicalis* SHC-03 was able to grow in phenol as the sole source of carbon and shift from carbohydrate metabolism to an enhanced phenol degradation pathway, as enhanced by the significant upregulation of genes encoding phenol 2-monooxygenase and catechol 1,2-dioxygenase. Phenol degradation rates were 99.5% and 95.9% at the phenol concentrations of 1.6 and 1.8 g/L, respectively.

Pesticides are widely used to control insect pests. However, their extensive application has raised concerns about environmental contamination. Faridy et al. isolated three bacterial consortia, FD, TD, and MD, from paddy soils. These species were predominant in the genera *Azospirillum, Ochrobactrum, Sphingobium*, and *Sphingomonas*. All three consortia were able to biodegrade more than 80% of fipronil (FIP), thiobencarb (THIO), or FIP + THIO in 10 days through oxidative and hydrolytic processes.

Conventional and biodegradable microplastics are widely distributed in oceans, freshwater, and sediments, potentially harming humans. Microbes can remediate microplastics through biodegradation. In nature, microplastics are colonized by biofilm-forming microbes, and different microplastics may host different microbes. Jiao et al. found that the majority of microbes colonizing conventional microplastics had nitrification and denitrification properties. In contrast, the dominant bacteria on biodegradable microplastics were able to degrade lignin, cellulose, and engage in carbon metabolism. The differences in bacterial communities were largely related to the intrinsic properties of the materials themselves, along with changes in the physicochemical attributes of the sediments.

Dinitrophenols (DNPs) are a class of synthetic organic compounds used in the manufacture of dyes and explosives. Soil-buried landmines containing DNP could be dangerous to personnel. Kim et al. studied the responses of the YhaJ transcription factor in *E. coli* strain K-12 to DNP and its metabolic products since a small amount of DNP byproduct vapor emitted from the landmines can be detected by Yhaj-based biosensors. By mutating the DNA-binding domain of Yhaj, a mutant known as S23R had increased biosensor capability for DNPs. This study highlights the importance of genetic manipulation of microbes in improving the efficiency of bioremediation.

In summary, microbes represent an affordable means of remediating environmental pollutants. The articles published in this Research Topic highlight recent progress in microbial remediation of heavy metals and organic compounds in water and soil. Reported mechanisms for heavy metal remediation mainly involve biosorption. Future research on the manipulation of ion transporters, the process of detoxification, ion chelation, and compartmentation should be emphasized. Microbial remediation of organic pollutants should also address novel biodegradation pathways and genetic engineering of key genes in the pathway to improve remediation efficiency.

